# On the link between shadow economy and carbon dioxide emissions: an analysis of homogeneous groups of countries

**DOI:** 10.1007/s11356-023-30385-z

**Published:** 2023-10-20

**Authors:** Nuno Silva, José Alberto Fuinhas, Masoud Shirazi

**Affiliations:** Faculty of Economics, CeBER, University of Coimbra, Coimbra, Portugal

**Keywords:** Shadow economy, Environmental Kuznets curve, Carbon dioxide emissions, Heterogeneous panels, Panel quantile regression, Panel smooth transition regression, Q53, Q56, C50

## Abstract

In the framework of an environmental Kuznets curve, the linkage between shadow economy and carbon dioxide (CO_2_) emissions was evaluated for 145 countries from 1991 to 2017. In assessing the effect of the shadow economy on CO_2_ emissions, we used panel quantile regression, panel fixed effects, and panel smooth transition regression as estimation methods. In addition, to deal with parameter heterogeneity, we resorted to the procedure of Lin and Ng (2012). We found two country groups that share homogeneous parameters. No environmental Kuznets curve was found for the set of all countries. Nevertheless, one was found for each of the homogeneous parameter country groups. This result supports different turning points for different groups of countries. Shadow economy contributed to reducing CO_2_ emissions in group 1 and aggravated it in group 2. Manufacturing was revealed to be statistically significant for the countries of group 1. Fossil fuel rents increased the CO_2_ emissions, mainly in group 2. Urbanization contributed to the hike of CO_2_ emissions in both country groups but much more intensely for group 1. Evidence of a tendency for decreasing CO2 emissions was also found, reflecting the efficiency gains over time.

## Introduction

Understanding the link between the shadow economy (informal or underground economy) and carbon dioxide emissions gains relevance as the climatic problem worsens. Indeed, researching this link helps quantify the extent of carbon dioxide emissions associated with informal economic activities, providing a more comprehensive picture of a country’s carbon footprint and allowing policymakers to design incentives and a proper intervention to achieve zero emissions. Comprehension of the connection between the shadow economy and CO2 emissions is crucial to contribute to global efforts to combat climate change while addressing social and economic aspects of the informal economy.

This work focuses on the environmental Kuznets curve (EKC) controlling for the shadow economy, using a broad panel of 145 countries from 1991 to 2017. The EKC posits an inverted U-shaped relationship between economic growth and environmental degradation. In the early stages of a country’s development, the need to increase material production to reduce poverty and hunger takes center stage, leading to increased pollutant emissions. Later, as incomes rise, people become more concerned about environmental protection, and governments establish institutions and enact laws to protect the environment, resulting in lower environmental pressures.

The shadow or non-observed economy refers to all economic activities hidden from official authorities. According to OECD (2014), these activities can be grouped into five broad classes: (i) underground production, which represents the legal product that is deliberately concealed from public authorities; (ii) illegal production; (iii) informal sector production; (iv) production of households for own-final use; (v) statistical underground, which refers to unrecorded production activities due to deficiencies in statistical systems.

The size of the shadow economy varies widely across countries and even over time. Therefore, there is no easy way to measure it. Nevertheless, several attempts have been made to obtain a value for the dimension of the phenomenon, such as the works of Elgin et al. (2021) and Medina and Schneider (2019).

The underground economy causes measurement errors in the true value of the economic activity, leading to biases in gross domestic product. This issue is particularly relevant as countries adopt diverse practices for estimating and including underground activities in measured gross domestic product (OECD 2012). Typically, developed countries attempt to estimate the shadow economy and include it in their gross domestic product. However, poorer countries often lack the will and the resources to do that. As a result, the shadow economy treatment often varies among countries at similar development levels.

In sum, our contribution to the literature on the EKC hypothesis involves the combination of several features that were not considered in past studies. First, we assess the EKC hypothesis for a large panel of 145 countries over 27 years, accounting for the heterogeneity problem in longitudinal data by forming homogeneous country groups based on the fixed-effects estimates. This innovation clarifies situations where EKC hypotheses do not hold, contributing to shed light on the Altman and Bland (1995) warn “absence of evidence is not evidence of absence.” Second, we include the shadow economy as an explanatory variable to control for the measurement errors in economic activity. Third, we use time as a proxy for technological evolution, which is crucial because late-developing countries can access modern and clean production techniques. Finally, we examine the stability of the marginal effects of the covariates on carbon emissions using the panel transition regression model.

The remaining of this study is organized as follows: “Literature survey, theoretical background, and research hypothesis” section surveys the literature. “Data and methods” section presents the data and the methods used. “Empirical results” section reveals the results. Finally, “Discussion” section discusses the results, and “Conclusion and policy implications” section presents the conclusion and policy implications.

## Literature survey, theoretical background, and research hypothesis

### Literature survey

Concerns about environmental degradation have led to the development of literature on the factors and policies that affect environmental quality, usually represented by CO_2_ emissions (Sultana et al. 2022; Shirazi and Šimurina 2022; Işık et al. 2021; Al-Mulali and Ozturk 2015). This section summarizes the recent studies on the determinants of environmental quality, including economic growth, energy consumption, and institutional arrangements in a jurisdiction, e.g., policies, regulations, acts, and informal economy, which are first summarized in Table 1. Then, the theoretical framework for linking the shadow economy, the controlling variables, and the ${\mathrm{CO}}_{2}$ emissions are presented. Finally, the research hypotheses are organized.

**Table 1:** Representative works on the determinants of environmental quality

Environmental quality and economic growth		
Author	Sample	Key Findings
Sultana et al. (2022)	Emerging economies	GDP $\uparrow \to$ environmental quality $\uparrow$
Ozcan et al. (2020)	OECD	GDP $\uparrow \to$ environmental degradation $\uparrow$
Danish and Wang (2019)	Next-11	GDP $\uparrow \to$ environmental degradation $\downarrow$
Rahman (2020)	Top 10 electricity-consuming countries	Validity of the EKC hypothesis
Erdogan et al. (2020)	OECD	Validity of the EKC hypothesis
Ahmad et al. (2022)	Emerging economies	Validity of the EKC hypothesis
Destek and Sarkodie (2019)	Emerging economies	Validity of the EKC hypothesis for Mexico, the Philippines, Singapore and South Africa; U-shaped relationship in China, India, South Korea, Turkey, and Thailand
Usman et al. (2021)	MENA	U-shaped relation
Halliru et al. (2020)	West Africa	U-shaped relation
Onifadea et al.(2021)	E7	GDP $\uparrow \to$ environmental degradation $\uparrow$
Environmental quality and energy consumption		
Author	Sample	Key findings
Sultana et al. (2022)	Emerging economies	Renewable energy consumption $\uparrow \to$ environmental degradation $\downarrow$ and non-renewable energy consumption $\uparrow \to$ environmental degradation $\uparrow$
Ahmad et al. (2021a); Djellouli et al. (2021)	African countries	
Khan et al. (2021)	OECD	
Pata (2021)	US	
Le and Sarkodie (2020)	EMDE	
Pata and Caglar (2021)	China	No contribution between renewable energy consumption and environmental degradation
Institutional arrangements and environmental quality		
Author	Sample	Key findings
Sultana et al. (2022)	Emerging economies	Corruption control upgrades environmental quality by reducing the ecological footprint
Ahmad et al. (2022)	Emerging economies	Institutional quality moderates the impact of financial development on the ecological footprint
Azam et al. (2021); Du (2021); Momtaz and Kabir (2018)	Developing countries	Depends on effectively enforcing the environmental rules and regulations
Du (2021); Ren et al. (2021); Dogan et al. (2020)	Developing countries	Illegal activities may cause ${\mathrm{CO}}_{2}$ emissions, water pollution, degraded land, and forest resources
Goel et al. (2013)	MENA	The negative impact of corruption on the pollution level
Environmental quality and informal economy		
Author	Sample	Key findings
Sultana et al. (2022); Köksal et al. (2020)	Emerging economies; Turkey	The long-run positive impact of informal economic activities on the environmental quality
Ortiz et al. (2022); Pang et al. (2021); Huynh (2020); Karanfil and Ozkaya (2007)	101 countries; China; developing Asia; Turkey	Informal economy causes environmental pollution
Zhang et al. (2020); Benkraiem et al. (2019)	China; Bolivia	Unrecorded economy lowers the environmental quality
Sohail et al. (2021); Bano et al. (2018); Nkengfack et al. (2021)	Sri Lanka and Nepal; Sub-Saharan Africa	Significantly negative nexus between the informal economy and ${\mathrm{CO}}_{2}$ emissions
Zhou et al. (2019); Elgin and Oztunali (2014)	China; global context	Inverted U-shaped relation between environmental pollution and the informal economy
Canh et al. (2021)	115 countries	Shadow economies are highly energy-intensive and lowly green-oriented
Chu (2022); Baksi and Bose (2016)	OECD; developing countries	Analyzes how the informal sector acts to outsource polluting production
Bento et al. (2018)	US	Energy tax can create welfare gains by allowing substitution from the informal sector

*OECD* Organization for Economic Co-operation and Development, *MENA* Middle East and North Africa, *E7* 7 major emerging countries, *Next-11* 11 newly industrialized countries, *EMDE* emerging markets and developing countries

As seen in Table 1, the studies on the relationships between environmental quality and the shadow economy are largely unknown and ignored, although the informal economy encompasses various pollution-intensive activities that intensify the environmental impacts (Wang et al. 2019). Remarkably, the existing literature mainly refers to fossil fuel consumption and GDP per capita as the main determinants of ${\mathrm{CO}}_{2}$ emissions (Adekoya et al. 2022; Ahmad et al. 2021b; Alvarado et al. 2018, among others). The studies commonly present results to assess whether the economies experience the EKC hypothesis (Churchill et al. 2018). Likewise, other determinants of environmental deterioration are resource rents, oil prices, industry value-added, urbanization, net inflow of foreign direct investment, trade openness, and technological advances (Adekoya et al. 2022; Su et al. 2022; Chandio et al. 2021; Ponce and Alvarado 2019).

Accordingly, most of the explanations for environmental degradation considered in the existing literature are associated with the activities through the formal economy. Therefore, this research explicitly focuses on the potential non-linear impact of the shadow economy on the ${\mathrm{CO}}_{2}$ emissions (Sultana et al. 2022; Köksal et al. 2020; Zhou 2019). Besides controlling for variables such as economic growth, manufacturing value-added, urbanization, and fossil fuel rents, the effect of technological progress on the ${\mathrm{CO}}_{2}$ emissions is also investigated. This contribution is handled by disentangling the direct impact of time on emissions from its indirect effects (González et al. 2005), which is rare in the recent literature.

### Theoretical background

#### Linking the shadow economy and the CO2 emissions

The role of factors related to the shadow economy, such as the informal economy, corruption, and deregulation in environmental pollution, has been studied by a limited number of articles, which is recognized as relevant and in need of innovative investigation (Ortiz et al. 2022; Chu 2022; Tillaguango et al. 2021; Shao et al. 2021).

The first strand of reviewed articles refers to the deregulation effect and concludes that the shadow economy increases pollution and damages environmental sustainability. Through the nexus between environmental regulation and pollution, the literature has explained some features of the shadow economy that underlie its relationship to environmental quality (Sultana et al. 2022; Ahmad et al. 2022, among others).

Through the nexus between environmental regulation and pollution, Ortiz et al. (2022) apply a panel data set of the “spatial autoregressive models,” “spatial lag models,” and “Durbin spatial models” and show that pollution has a large spatial dependence on environmental pollution processes. Pang et al. (2021) first utilize the “Multiple Indicators Multiple Causes” technic to calculate the shadow economy. Then, they used the “Generalized Method of Moments” and found a statistically significant bidirectional positive relationship between the shadow economy and pollution across 31 provinces of China from 2005 to 2015.

From the other viewpoint, Canh et al. (2021) emphasize that informal economic activities are less environmentally friendly and more energy-intensive, given that environmental rules and regulations are largely ignored. They indicate that compliance with the official requirements is stronger in the formal sector than in the informal sector. Hence, the formal sector avoids costly rules and regulations by outsourcing parts of the production process to the informal sector. Also, Baksi and Bose (2016) identify that the informal sector can act as an origin of leakage by allowing the formal sector to facilitate the outsourcing of polluting production.

From another perspective, Bento et al. (2018) show that energy taxes may create welfare gains through easing substitution from the shadow economy. They mention two mechanisms by which the informal sector may lower the costs of energy taxes and environmental policies: (i) energy taxes impose indirect costs on the informal sector as informal firms order energy sources through the formal sector, e.g., natural gas, electricity, and gasoline, and (ii) a revenue-neutral shift in tax bases toward energy sources that could mitigate the tax burden on the goods substituted via the informal sector. These mechanisms are determined to have remarkable potential in the welfare-improving substitution from the informal sector to the formal sector. Accordingly, a well-judged policy portfolio is required to reduce the damaging effect of the informal sector on environmental quality.

The second category of recent studies is associated with the scale, composition, and technique effects mechanisms where the underground economy is related to environmental pollution (Chu 2022; Sohail et al. 2021; Nkengfack et al. 2021).

In this respect, Chu (2022) employs an “advanced panel quantile regression” to study the effect of the shadow economy on the ecological footprint of the OECD economies from 1995 to 2015. The empirical findings indicate that due to the composition and technique effects, the shadow economy negatively impacts the mid to high quantiles of the ecological footprint. Furthermore, technological innovation exerts a favorable effect on lowering the ecological footprint, but it is non-monotonic across quantiles. The study concludes that higher informality mitigates the favorable impact of technological innovation on environmental quality.

In a group of countries with highly informal economies and as a consequence of the composition effect, Sohail et al. (2021) found a significantly negative nexus between the informal economy and ${\mathrm{CO}}_{2}$ emissions in Nepal and Bangladesh. However, the beneficial and detrimental role of the informal economy is suggested through a non-linear relationship in Sri Lanka and Nepal, respectively. Also, Nkengfack et al. (2021) find that higher informal activities in Sub-Saharan African countries with lower middle income reduce the ${\mathrm{CO}}_{2}$ emissions in the long run.

In conclusion, the nexus between the shadow economy and environmental degradation can be non-monotonic when associated with the pollution level. Besides, taking into account the two distinct impacts of informality, it is required to investigate the comprehensive effect of the shadow economy on the ${\mathrm{CO}}_{2}$ emissions, especially from the left to the right tail of the emissions’ conditional distribution. To this end, the impacts of explanatory variables on low, medium, and high quantiles of ${\mathrm{CO}}_{2}$ emissions are examined in this paper by the “panel quantile regression model” (Canay 2011). Furthermore, the evolution of the covariates’ slopes over time is analyzed by the “panel smooth transition regression model” (González et al. 2005).

#### Linking the controlling variables and the CO_2_ emissions

In parallel, there is a growing literature that examines the role of economic growth on ${\mathrm{CO}}_{2}$ emissions (Sultana et al. 2022; Ahmad et al. 2022; Onifadea et al. 2021, among others). In this regard, Li and Wei (2021) use the panel smooth transition regression method (PSTR) and indicate the existence of non-linear relationships between economic growth, innovation, trade openness, financial development, and carbon emissions.

Wang and Wei (2020) also apply the PSTR method and show that emerging economies associate with a strict level of environmental regulation, which will cause serious “green paradox” effects and delay economic development. Furthermore, they illustrate that the rebound effects of technology on energy consumption increase the CO2 emissions in the OECD countries. Notably, technological improvements enhance economic growth and promote efficiency, decrease the cost of energy consumption, and, therefore, increase energy usage, which is called the rebound effect (Lin and Du 2015). Panayotou (2016) utilizes a set of “reduced form single-equation” models and shows that the potential inverted U-shaped relationship is justified through the “scale, composition, and technique effects.”

Particularly, scholars underscore that economic growth requires more input volume, producing more natural resource usage. In other words, during the inefficient industrialization process, the environmental consequences are neglected, which refers to the scale effect and leads to increasing pollution (Ahmed et al. 2020a; Zafar et al. 2019; Banday and Aneja 2019). However, as economic growth increases, the structure of an economy changes called the composition effect, which gradually expands the cleaner activities, producing lower pollution (Dinda 2004) due to more efficient human capital accumulation. At high levels of economic growth, the underdeveloped and developing technologies are replaced by the developed and cleaner technologies, called the technique effect, which can enhance the environmental quality (Bano et al. 2018).

Concerning other controlling variables, urbanization and industrialization require a higher volume of inputs that leads to more natural resource usage in both consumption and production activities (Langnel and Amegavi 2020), which causes more natural resource extraction (Ahmed et al. 2020a) and ${\mathrm{CO}}_{2}$ emissions (Su et al. 2022; Langnel et al. 2021; Ullah et al. 2021; Ahmed et al. 2020b; Danish 2019).^1^

Finally, most studies argue that postponing the switch from the carbon-based fuel portfolio to clean sources does not reduce fossil fuel consumption and lowers the ${\mathrm{CO}}_{2}$ emissions, suggesting the resource rents raise the environmental degradation (Adekoya et al. 2022; Ali et al. 2021; Langnel et al. 2021; Shahzad et al. 2021; Ullah et al. 2021; Ebrahimi Salari et al. 2021; Nathaniel and Khan 2020; Zafar et al. 2020; Ahmed et al. 2020a,b; Destek and Sinha 2020; Danish 2019; Alola et al. 2019; Ozcan et al. 2019; Li and Sun 2018; Ulucak and Lin 2017). On the other hand, Ulucak and Khan (2020) reveal that renewable energy and resource rents reduce the ecological footprint across the BRICS economies, suggesting their interesting role in reducing environmental degradation.

From the survey of the reviewed literature, it is detected that the contradictions remain in the nexus between the controlling variables, such as economic growth, urbanization, technological progress (Trend), fossil fuel rents, and environmental degradation, which necessitate further investigation. Accordingly, this study fills in the gap and aims to explore new insights by considering homogeneous groups of countries to derive relevant policy implications adapted to the different groups that promote their sustainable economic growth with a low environmental cost.

### Research hypothesis

Underlying gross domestic product measurement errors lead to biases in assessing the relationship between economic activity and environmental degradation. This problem is particularly acute for less developed countries, where these measurement errors tend to be higher. Thus, it is crucial to include estimates of the underground economy when testing the EKC hypothesis, which leads us to our first hypothesis.

- Hypothesis 1. The shadow economy affects carbon dioxide emissions, and we expect it to cause an increase in emissions for poorer countries for which gross domestic product measurement errors are typically high.

To have a big picture of the role of the shadow economy in environmental degradation behavior, the researchers can use large panels of countries. However, the indiscriminate inclusion of countries in panel estimations leads to huge heterogeneity that blurs the quality of these estimations, allowing conclusions that do not cope well with the idiosyncratic features of countries. The computationally intensive econometric technique proposed by Lin and Ng (2012) was used to circumvent the problem of heterogeneity in longitudinal data, which allows for identifying groups with homogeneous parameters. Indeed, this technique can be used to identify groups of countries with homogeneity of parameters for an econometric specification made a priori.

A proper assessment of the EKC hypothesis requires the inclusion of control variables that account for the different characteristics of countries. Otherwise, we might incur an omitted variables problem that leads to biased estimates and erroneous conclusions. We choose manufacturing value-added as a percentage of GDP, the urbanization rate, and fossil fuel rents as a portion of GDP as control variables. We expect higher urbanization and industrialization to translate into higher CO2 emissions, as production activities require more inputs and resources. Regarding fossil fuel rents, we conjecture that countries that rely heavily on fossil fuels have few incentives to promote their transitions to renewable energy sources. Thus, we expect their pollution levels to be higher.

- Hypothesis 2. Manufacturing value-added as a percentage of GDP, the urbanization rate, and fossil fuel rents as a fraction of GDP contribute to raising CO_2_ emissions.

Another neglected factor in prior studies focusing on the EKC hypothesis is time. Past research implicitly assumes that countries that share the same values for GDP and other control variables are expected to have the same level of CO_2_ emissions. In our view, this conjecture is incorrect, as late-developing countries benefit from more advanced and clean technologies that allow them to achieve the same GDP level while polluting less. This fact leads us to formulate our third hypothesis.

- Hypothesis 3. Time, as a proxy for technological development, mitigates carbon dioxide emissions, given the development level of countries.

Time may also influence the linkages between the explanatory variables and carbon dioxide emissions. As technology evolves, the marginal impact of manufacturing, urbanization, and other variables on emissions may change. Thus, we resort to the panel smooth transition regression model, proposed by González et al. (2005), to evaluate whether the marginal effects of the regressors on emissions are time varying.

- Hypothesis 4. The marginal effects of the explanatory variables on carbon dioxide emissions change over time.

## Data and methods

In this section, the data and method are explained in detail.

### Data

In this research, we select the annual carbon dioxide emissions in metric tonnes per capita (*CO2*), retrieved from the World Bank Open Data (2021), as our measure of environmental degradation. The extensive use of this variable in studies focusing on the environmental Kuznets curve motivates our choice. We consider several drivers of emissions, listed in Table 2.

**Table 2:** Data description

Variable	Description	Source
CO2	Carbon dioxide emissions in metric tonnes per capita	World Bank Open Data (2021)
GDP	Gross domestic product per capita in constant 2010 USD	World Bank Open Data (2021)
GDP2	Squared gross domestic product per capita in constant 2010 USD	World Bank Open Data (2021)
Shadow	A measure of the size of the unobserved economy in constant 2010 USD. It is calculated by multiplying estimates of the shadow economy as a fraction of gross domestic product by gross domestic product	Medina and Schneider (2019)
Manuf	Manufacturing value-added as a percentage of gross domestic product. Manufacturing refers to industries in ISIC divisions 15–37	World Bank Open Data (2021)
Rent	Fossil fuel rents as a percentage of gross domestic product. The sum of oil, coal, and natural gas rents as a portion of the gross domestic product	World Bank Open Data (2021)
Urban	Urbanization rate. The urban population is a percentage of the total population	World Bank Open Data (2021)

The availability of the shadow economy variable drove our choice of the list of countries under study and the period. Medina and Schneider (2019) define the shadow or informal economy as all economic activities hidden from official authorities for monetary, regulatory, and institutional reasons. The unobserved economy is noticeably difficult to measure, as the entities that engage in these activities want them to remain unnoticed. In general terms, there are two different types of methods to assess the size of the informal economy: direct approaches and indirect approaches. The first type relies on surveys and samples, and their results are sensitive to the manner in which the questions are formulated and the willingness of the respondents to reveal their activities, rendering them unreliable truthfully.

Indirect methods resort to indicators to assess the size of the shadow economy, such as the difference between national expenditure and income, the difference between the actual and official labor force, and electricity consumption, among others. Medina and Schneider (2019) use the “Multiple Indicators, Multiple Causes” method (MIMIC), which is more robust than single indicator methods, as it considers several causes for the extent of the shadow economy and various effects. This method links the observable causes to their effects on an unobservable variable to estimate the size of the shadow economy. Specifically, they consider eight causes for the informal economy (trade openness, GDP per capita, unemployment rate, size of government, fiscal freedom, rule of law, control of corruption, and government stability) and three effects (currency as a fraction of broad money, labor force participation, and the growth rate of GDP per capita). Using the MIMIC method with the causes and effects described above, these authors obtain annual estimates for the shadow economy covering 157 countries from 1991 to 2017. We excluded twelve countries that did not have information on all the variables pertinent to this study for at least 10 years. Thus, our final sample includes annual data for 145 countries from 1991 to 2017.

**Fig. 1:**
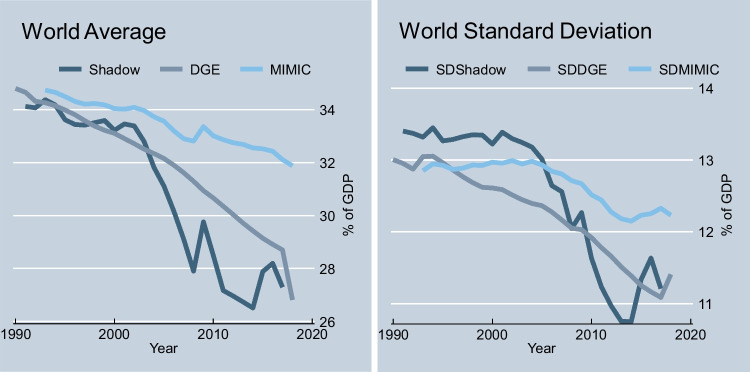
Three measures of the shadow economy. Shadow denotes the size of the shadow economy in % of GDP as estimated by Medina and Schneider (2019); DGE denotes dynamic general equilibrium model-based (DGE) estimates of informal output (% of official GDP), and MIMIC multiple indicators multiple causes model-based (MIMIC) estimates of informal output (% of official GDP), both as estimated by Elgin et al. (2021); this figure was created by the authors based on the data from Medina and Schneider (2019) and Elgin et al. (2021)

Figure 1 reveals the world mean and standard deviation of three shadow/informal economy measures estimated by Medina and Schneider (2019) and Elgin et al. (2021). One can see a trend for decreasing the average and the dispersion in the phenomena. The decoupling of estimates starts with the event of the financial crisis of 2007 and is more pronounced for the estimates (Shadow) of Medina and Schneider (2019) than for the ones (DGE and MIMIC) of Elgin et al. (2021).

Table 3 shows the descriptive statistics for all the countries in the sample, group 1 and group 2. The following section describes the method used to create the country groups. Finally, in Appendix A, we offer the complete country list and groups 1 and 2 composition. Curiously, even though the country selection procedure is based exclusively on the data and includes no aprioristic view about the groups’ compositions, despite its smaller size (42 countries in group 1 versus 103 in group 2), group 1 includes most countries of the European Union plus the UK, and the largest developed economies, such as the US and Japan.

**Table 3:** Descriptive statistics

Variables		Obs	Mean	Std.-dev	Min	Max
CO2	FS	3912	4.662	5.872	0.016	47.700
	G1	1134	7.549	6.281	0.588	47.700
	G2	2778	3.484	5.261	0.016	31.748
GDP	FS	3842	12.966	18.398	0.164	111.968
	G1	1098	21.004	18.783	0.450	71.296
	G2	2744	9.750	17.224	0.164	111.968
GDP2	FS	3842	506.514	1268.359	0.027	12,536.91
	G1	1098	793.644	1042.765	0.202	5083.132
	G2	2744	391.621	1331.025	0.027	12,536.91
Shadow	FS	3842	2.394	2.588	0.057	19.578
	G1	1098	3.412	2.492	0.096	12.079
	G2	2744	1.987	2.513	0.057	19.578
Manuf	FS	3641	13.756	6.264	0.000	50.037
	G1	1026	14.769	6.661	0.233	44.599
	G2	2615	13.358	6.056	0.000	50.037
Rent	FS	3844	4.284	9.642	0.000	58.982
	G1	1117	8.207	13.335	0.000	55.593
	G2	2727	2.677	7.026	0.000	58.982
Urban	FS	3915	56.153	23.223	5.491	100
	G1	1134	67.571	19.832	20.629	100
	G2	2781	51.497	22.893	5.491	100

This table was built using the Stata function *summarize*. FS, G1, and G2 identify the full sample, group 1 and group 2, respectively. Obs. denotes the number of observations; Std.-dev. denotes the standard deviation; Min. and Max. denote minimum and maximum, respectively; the command *sum* of Stata was used

The average per capita CO_2_ emissions are substantially higher for group 1 countries than for group 2. The first country group is wealthier, and the size of its informal economy, measured in constant 2010 USD, is higher. However, its average size relative to the gross domestic product is lower (16.24% and 20.38% for groups 1 and 2, respectively). Group 1 countries have a more urban population and a slightly higher gross domestic product and manufacturing share. There is a sizable difference regarding fossil fuel rents, those of the first group more than tripling of the second group.

### Methods

We begin this subsection by detailing preliminary tests to assess the data properties. Then, we present the main model and estimation methods: (i) Canay (2011) panel quantile regression, (ii) panel fixed effects, and (iii) panel smooth transition regression (González et al. 2005). Finally, we describe the procedure proposed by Lin and Ng (2012) to deal with the potential problem of parameter heterogeneity, which involves forming country groups based on the fixed-effects estimates.

#### Preliminary tests

We perform the following tests to check the properties of the variables used in our analysis:

(I) The Shapiro–Wilk normality test (Royston 1983). This test verifies whether the variables are normally distributed. The null hypothesis states they are.
(II) The variance inflation factor (VIF) is used to evaluate the degree of multicollinearity between the explanatory variables. High multicollinearity may render parameter estimates unstable.
(III) Cross-sectional dependence test (Pesaran 2021). According to the null hypothesis, there is no correlation between different units. If this hypothesis does not hold, the traditional fixed effects estimator is inefficient, and the standard errors are biased.
(IV) Panel unit root test (CIPS) analyzes the stationarity of the variables (Pesaran 2007). The null hypothesis of this test states that the series is non-stationary. Spurious regression is a common consequence of using integrated variables.
(V) Cointegration test (Pedroni 1999). According to the null hypothesis, variables are not cointegrated. A set of integrated variables of order one is cointegrated if a linear combination of these variables is stationary. Cointegration avoids the spurious regression problem and renders parameter estimates super-consistent.
(VI) The Hausman test (Hausman 1978) compares the fixed and random effects estimates. Under the null hypothesis, the random effects estimator is consistent and efficient. However, if the unobserved effect is correlated with the independent variables, the null hypothesis fails, and the fixed effects estimator should be chosen.

#### Panel quantile regression

Next, we proceed to the estimation stage. We resort to panel quantile regression as our main estimation method. This method has several advantages compared to traditional least-squares: (i) it is robust in the presence of outliers, which may generate large swings in least-square parameter estimates; (ii) it works well with non-normally distributed data; and (iii) we can get a thorough image of the impact of the carbon emission’ drivers on all its distribution because, unlike least-square methods, quantile regression does not model the conditional mean exclusively.

We conjecture the relationship between per capita carbon dioxide emissions and their drivers following the specification below:

1 $${CO2}_{i,t}={{\alpha }_{i}+X}_{i,t}^{\prime}\beta \left({U}_{i,t}\right)$$

where the index $i=\mathrm{1,2},\dots ,145$ identifies the country; $t=\mathrm{1991,1995},\dots ,2017$ corresponds to the observation year; ${\alpha }_{i}$ is the country’s fixed effect; ${X}_{i,t}{\prime}=\left[{1, GDP}_{i,t}, {GDP2}_{i,t}, {Shadow}_{i,t}, {Manuf}_{i,t}, {Rent}_{i,t},{Urban}_{i,t},{trend}_{t}\right]$ is the explanatory variables’ vector; $\beta \left({U}_{i,t}\right)=\left({\beta }_{0},{\beta }_{1},\dots ,{\beta }_{7}\right)$ is the coefficients’ vector; and ${U}_{i,t}$ is a uniformly distributed random variable on the interval $\left[\mathrm{0,1}\right].$

The relationships between CO_2_ emissions and economic growth, the share of manufacturing in GDP, and urbanization are extensively documented in the literature. However, including the remaining covariates in our model deserves further justification. First, countries that draw high rents from fossil fuels may be unwilling to invest in environmentally friendly technologies and renewable energy sources. They have vast non-renewable energy resources to ensure their energetic security and develop their economies. Thus, we expect the variable Rent to affect CO2 emissions positively. The inaccuracy of gross domestic product explains the inclusion of the shadow economy among the covariates as a measure of the true level of economic activity. Even though countries should include all non-observable economic activities in their GDP estimates, some choose not to.^2^ Many others do not exhaustively measure the shadow economy^3^ (United Nations Economic Commission for Europe 2008; Gyomay 2014). Also, the least developed countries often lack the means and/or the willingness to obtain reliable estimates of the underground economy. Finally, we believe time is a very important and often neglected variable in environmental Kuznets curve studies. The traditional approach to the environmental Kuznets curve assumes that two countries that share the same characteristics (GDP and other control variables) in two different years should have the same carbon dioxide emissions. This conjecture is incorrect, as late developers have access to innovative and eco-friendly technologies. Furthermore, the rising worldwide pressure to reduce greenhouse gas emissions to mitigate global warming is likely to drive a decrease in carbon emissions in the most recent years, *ceteris paribus*. Thus, we expect the passage of time to contribute to decreasing emissions.

Simple quantile regression may lead to inconsistent parameter estimates in Eq. (1) when the independent variables are correlated with the unobserved fixed effects. Canay (2011) designed a simple two-step estimation procedure to overcome this problem. Let ${u}_{i,t}\equiv$ ${X}_{i,t}^{\prime}\left[\beta \left({U}_{i,t}\right)-{\beta }_{\mu }\right]$, where ${\beta }_{\mu }$ is the conditional mean of $\beta \left({U}_{i,t}\right)$. From Eq. (1), we get

2 $${CO2}_{i,t}={{\alpha }_{i}+X}_{i,t}{\prime}{\beta }_{\mu }+{u}_{i,t}$$

The method proposed by Canay to estimate the quantile *τ*’s parameters proceeds as follows:

1. Get a consistent estimate of ${\beta }_{\mu }$, using Eq. (2), and let ${\widehat{\alpha }}_{i}\equiv {T}^{-1}\sum\limits_{t=1}^{T}\left[{CO2}_{i,t}-{X}_{i,t}^{\prime}{\widehat{\beta }}_{\mu }\right]$, where ${\widehat{\beta }}_{\mu }$ is the estimate of ${\beta }_{\mu }$.
2. Estimate the coefficients of the explanatory variables for quantile *τ* by solving the following problem:
  3 $$\widehat{\beta }\left(\tau \right)\equiv {argmin}_{\beta }\frac{1}{T\times N}\sum\limits_{t=1}^{T}\sum\limits_{i=1}^{N}\left[{\rho }_{\tau }\left({\widehat{CO2}}_{i,t}-{X}_{i,t}^{\prime}\beta \right)\right]$$

where ${\widehat{CO2}}_{i,t}={\mathrm{CO}2}_{i,t}-{\widehat{\alpha }}_{i}$, ${\rho }_{\tau }$ is the check function for quantile *τ*, *T*, and *N* denote the number of years and the number of countries in our sample, respectively. Canay (2011) shows that the estimates obtained using this method are consistent and asymptotically normal. Therefore, we use the fixed-effects estimates in the first stage of our application. Then, we compute the standard errors through a simple bootstrap procedure involving 1000 replications.

We compare the fixed-effects estimates for Eq. (1) with the quantile regression results.

#### Panel smooth transition regression

After confirming the impact of time on carbon dioxide emissions, we dig further into the analysis and study the channels through which time affects emissions. Note that it may change the sensitivity of emissions to the covariates. As technology advances, manufacturing may become less carbon-intensive, and improving building techniques may make dwellings more energy-efficient, thus weakening the linkage between these variables and emissions. Assessing the evolution of the GDP coefficients is also interesting, as late-developing countries may follow different paths to achieve the same level of well-being as early developers.

Beyond the direct effect of time on carbon dioxide emissions, it may also affect the strength of the linkages between the covariates and CO2. That is, as technology evolves and populations increase their demands for a reduction in emissions, the marginal effect of manufacturing and urbanization on emissions may diminish as industries and construction change to cleaner production techniques. To assess the indirect impact of time on emissions from its indirect effects, we resort to the panel smooth transition regression (PSTR) model (González et al. 2005). We may see this model as a generalization of the panel threshold regression (PTR) model developed by Hansen (1999), which allows for abrupt changes in the regression coefficients that depend on the value of an observed variable. However, the PSTR model is a nonlinear homogeneous panel model that contemplates rough parameter changes contrary to Hansen’s PTR. In this research, we use the following PSTR specification:

4 $${CO2}_{i,t}={{\alpha }_{i}+X}_{i,t}^{\prime}{\beta }_{0}+\sum\nolimits_{j=1}^{r}{\mathrm{X}}_{i,t}^{\prime}{\beta }_{j}{g}_{j}\left({q}_{i,t}^{\left(j\right)};{\gamma }_{j};{c}_{j}\right)+{u}_{i,t}$$

where ${X}_{i,t}^{\prime}$ is the covariates vector for country *i* in year *t*; ${\alpha }_{i}$ denotes the fixed individual effect for country *i*; ${\beta }_{0}$ is the constant part of the coefficients’ vector for the covariates; ${\beta }_{j}$ is the coefficients’ vector corresponding to the transition function *j*; ${g}_{j}\left({q}_{i,t};{\gamma }_{j};{c}_{j}\right)=\left[1+\mathrm{exp}\left(-{\gamma }_{j}\left({q}_{i,t}^{\left(j\right)}-{c}_{j}\right)\right)\right]$ is the logistic transition function *j*; ${q}_{i,t}$ is the corresponding transition variable; and ${\gamma }_{j}$ and ${c}_{j}$ are the slope and location parameters for the transition function *j*, respectively. Next, we select the trend as the transition variable, allow for a maximum of three regimes (*r* = 2), and estimate Eq. (4) by nonlinear least squares using the MATLAB code provided by Fouquau et al. (2008) and Colletaz and Hurlin (2006). Finally, we choose the optimal number of regimes using the homogeneity and the no remaining heterogeneity tests (see González et al. (2005) for further details).

#### Lin and Ng (2012) estimation procedure

Parameter heterogeneity is a problem that plagues most macro panels with a large number of countries, such as ours and compromises the validity of the estimates. It is well known that ignoring parameter heterogeneity in a panel data framework leads to inconsistent parameter estimates (Campello et al. 2019). A method to circumvent this problem is to assume complete parameter heterogeneity. However, this solution turns it into a time series estimation that forfeits the benefits of the panel data structure. Lin and Ng (2012) propose an alternative solution, which assumes there are various groups of countries with homogeneous slope parameters within each group but allows for slope heterogeneity for different groups. This method establishes a compromise between full parameter homogeneity and full parameter heterogeneity and maintains the advantages of using a panel data structure without suffering from the estimation bias that afflicts fixed-effects estimates for the full sample. The procedure designed by these authors takes an agnostic view of the number and composition of the groups and uses a modified K-means algorithm to attain conditional clustering. First, the algorithm requires choosing the number of groups, G, and the random assignment of all countries to one of the groups. Then, we repeat the following two stages until convergence is achieved:

1. Estimate the fixed effects slope coefficients, ${\beta }_{g}$, for each group.
2. Assign country *i* to group *g'*, where *g'* is the solution to the following minimization problem:

5 $$g'={argmin}_g\sum\nolimits_{t=1}^T\left[{C\overset\dots O2}_{i,t}-{\overset\dots{X'}}_{i,t}{\widehat\beta}_g\right]^2$$

where ${C\overset\dots O2}_{i,t}$ and ${\overset\dots{X'}}_{i,t}$ are the demeaned dependent variable and independent variables vector for the country *i* in year *t*, respectively, and ${\widehat{\beta }}_{g}$ denotes the estimate of the slope for group *g*. Step 2 must be applied to every country in the sample.

In this study, we consider a maximum of five groups and repeat the algorithm described above one million times for each choice of G, as Lin and Ng (2012) show that the final estimates are sensitive to the initial group allocations. Then we compute the following modified BIC criterion for each replication and number of groups (one million times five, which equals five million):

6 $$BIC\left(\widetilde{G}\right)=ln\left[\frac{1}{NT}{\sum }_{g=1}^{\widetilde{G}}\sum\nolimits_{i\in {I}_{g}}\sum\nolimits_{t=1}^{T}{\left[{C\overset\dots{O2'}}_{i,t}-{\overset\dots{X'}}_{i,t}^{\prime}{\widehat{\beta }}_{g}\right]}^{2}\right]+\widetilde{G}K\frac{{c}_{NT}ln\left(NT\right)}{NT}+\left(\widetilde{G}-1\right)\frac{ln\left({N}^{2}\right)}{{N}^{2}}$$

where *K* denotes the number of regressors, ${c}_{NT}=\sqrt{min\left(N,T\right)}$, and ${\widehat{\beta }}_{g}$ is the vector of estimates for group *g*. Finally, we choose the group compositions and corresponding estimates that minimize this criterion.

#### Robustness check

Panel data estimates are sensitive to outliers, which may produce distorted estimates and lead to erroneous conclusions. To assess whether outliers drive our results, we control for their presence by introducing dummy variables and repeating all the estimations. We consider an observation an outlier whenever the estimated residual is more than three standard deviations away from the mean.

## Empirical results

In the first part of this section, we show the results of the preliminary tests.^4^ Then, we analyze the fixed effects and quantile regression estimates. Finally, in the last part, we present a figure that depicts the time variation of the parameter estimates implied by the PSTR model.

### Preliminary tests

Table 4 shows the Shapiro–Wilk test for normality. We reject the null hypothesis of normality for all variables. Furthermore, our primary method (quantile regression) is robust even when the data distribution is not normal. Thus, the absence of normality is not a problem.

**Table 4:** The Shapiro–Wilk test for normality

Variables	Obs	*W*	*V*	*Z*	Prob > *z*
CO2	3912	0.745	554.859	16.451	0.0000
GDP	3842	0.706	629.920	16.771	0.0000
GDP2	3842	0.449	1180.364	18.405	0.0000
Shadow	3842	0.802	422.873	15.734	0.0000
Manuf	3641	0.967	66.416	10.898	0.0000
Rent	3844	0.581	896.897	17.691	0.0000
Urban	3915	0.977	50.391	10.205	0.0000

This table was built using the Stata function *swilk*

All variance inflation factors (Table 5) are lower than 10, except for *GDP*. This finding is unsurprising: by construction, we should expect a high correlation between *GDP* and *GDP2*. Moreover, the mean–variance inflation factor is well below the commonly accepted threshold of six. Thus, we need not be concerned that serious multicollinearity affects our estimates.

**Table 5:** Variance inflation factor

Variables	VIF	1/VIF	Mean VIF
GDP	17.16	0.058	
GDP2	9.16	0.109	
Shadow	4.72	0.212	
Manuf	2.45	0.408	2.56
Rent	1.14	0.877	
Urban	1.11	0.898	
Trend	1.07	0.932	

This table was built using the Stata function *vif*

Cross-sectional dependence is a common feature of all variables (Table 6). This result is hardly surprising. The rising interdependence between economies implies that shocks propagate swiftly across the world. An undesirable consequence is that standard errors from the regular fixed effects estimator are biased. Thus, we resort to Driscoll and Kray’s (1998) standard errors in the fixed-effects estimates and bootstrapped standard errors in the panel quantile regressions.

**Table 6:** The Pesaran CD test

Variables	CD-test	*p*-value	Corr	Abs (corr)
CO2	37.807	0.000	0.07	0.51
GDP	312.24	0.000	0.60	0.73
GDP2	309.122	0.000	0.59	0.72
Shadow	127.793	0.000	0.24	0.54
Manuf	73.397	0.000	0.15	0.49
Rent	113.42	0.000	0.22	0.26
Urban	273.506	0.000	0.52	0.83

This table was built using the Stata function *xtcd*

Cross-sectional dependence affects the size and power of the first-generation panel unit root tests. Therefore, we choose to test the order of integration of the variables through the Pesaran (2007) panel unit root test robust to this feature. In the specification without a trend, only the variable *Manuf* is stationary, whereas when a trend is included, none is (Table 7).

**Table 7:** Panel unit root test (CIPS test)

Variables	Panel unit root test (CIPS) (Zt-bar)			
	Without trend			With trend
	Lags	Zt-bar		Zt-bar
CO2	1	5.569		0.426
GDP	1	5.269		1.136
GDP2	1	10.462		5.596
Shadow	1	3.912		0.562
Manuf	1	− 5.090***		0.116
Rent	1	6.806		9.173
Urban	1	3.193		4.014

This table was built using the Stata function *multipurt*. The null hypothesis for CIPS stipulates that the series have a unit root

^*** ^denotes a statistically significant rejection at the 1% level

When the variables are not stationary, regression methods may find fallacious relations between the variables driven by their trending nature. This phenomenon, known as spurious regression, may lead to erroneous conclusions about the impact of the covariates on the dependent variable. This problem is absent when the non-stationary variables are co-integrated when a linear combination of the variables is stationary. In this scenario, the estimates are super-consistent (Hamilton 1994). Therefore, we use the Pedroni (1999) cointegration test to ensure that our estimates are reliable. All versions of this test reject the null hypothesis of no cointegration (Table 8). Thus, we do not need to worry about drawing wrong inferences from our estimates because the errors are stationary.

**Table 8:** Pedroni cointegration test

Estimator	Statistic	*p*-value
Modified Phillips-Perron test	7.9692	0.0000
Phillips-Perron test	− 11.4716	0.0000
Augmented Dickey-Fuller test	− 11.3791	0.0000

This table was built using the Stata function *xtcointtest*

Finally, the Hausman test (Table 9) strongly rejects the null hypothesis that the random effects estimator is consistent. Thus, we must resort to the fixed effects estimator.

**Table 9:** Hausman test

Test distribution	Statistic	*p*-value
Chi-squared (7)	197.45	0.0000

This table was built using the Stata function *hausman*

### Fixed effects and panel quantile regression

Table 10 displays the full sample estimation results. The fixed-effects estimates for the gross domestic product do not contradict the environmental Kuznets curve hypothesis, but they do not offer strong support either: the coefficients have the expected sign but are not statistically significant. The shadow economy increases CO_2_ emissions, which confirms hypothesis 1. The manufacturing share, fossil fuel rents, and urbanization also raise CO_2_ emissions (hypothesis 2), confirming our conjecture that countries with vast fossil fuel resources have fewer incentives to invest in green technologies and renewable energy. The passage of time contributes to the mitigation of environmental degradation, driven by the adoption of innovative clean technologies and the increasing pressure to combat global warming (hypothesis 3). The quantile regression estimates for the gross domestic product confirm the hump-shaped relation between emissions and economic growth in the two highest quantiles. The remaining coefficients’ signs are coherent with the fixed-effects estimates, except for *Rent* in the 10th quantile. The impact of *GDP*, the shadow economy, manufacturing share, urbanization, and fossil fuel rents on *CO2* increases as we move from the left to the right tail of the emissions’ conditional distribution. On the contrary, the mitigating effect of the trend of environmental degradation is stronger in the middle quantiles.

**Table 10:** Estimates for_CO_2_

Variables	Quantiles					OLS
	10th	25th	50th	75th	90th	Fixed effects
GDP	0.0138	0.0116	0.0327	0.0461*	0.0546**	0.0241
GDP2	− 0.0007**	− 0.0006*	− 0.0007**	− 0.0008***	− 0.0007*	− 0.0006
Shadow	0.7345***	0.7846***	0.7875***	0.8145***	0.7991***	0.8049***
Manuf	0.0361***	0.0406***	0.0414***	0.0444***	0.0562***	0.0498***
Rent	− 0.0002	0.0271***	0.0402***	0.0595***	0.0905***	0.0444***
Urban	0.0612***	0.0632***	0.0636***	0.0635***	0.0651***	0.0634***
Trend	− 0.0185***	− 0.0199***	− 0.0214***	− 0.0198***	− 0.0188***	− 0.0210**
Constant	− 1.5789***	− 1.5809***	− 1.4919***	− 1.4545***	− 1.5137***	− 1.5897***

The Stata function *xtscc* was used for the fixed-effects estimates, and the Stata functions *xtreg* and *qreg* were used in the first and second steps of the quantile regression estimation

^***^, **, and * denote statistical significance at the 1%, 5%, and 10% levels, respectively

The split of countries into two groups (Table 11) uncovers the hump-shaped pattern between CO_2_ emissions and economic growth characteristic of the environmental Kuznets curve, which was concealed by parameter heterogeneity in the complete sample. However, the turning points of emissions, 41,555 USD for group 1 and 72,062.5 USD for group 2, are well above the current *GDP* of most countries. We can also observe that the CO_2_ emissions of countries from group 1 are more sensitive to economic growth than those of group 2. The differing impact of the unobserved economy on CO_2_ for the two groups—negative for group 1 and positive for group 2—deserves further study. The remaining coefficients agree with the complete sample estimates, except for the manufacturing coefficient for group 2. The effects of manufacturing and urbanization on CO_2_ emissions are stronger for group 1 than group 2, while the reverse happens for fossil fuel rents. Combining these two later results is evidence of the resource curse for group 2 (e.g., Fuinhas and Marques 2013). The passage of time mitigates emissions the most for group 1. The quantile regression estimates agree with the fixed-effects estimates in sign and significance, except for *Rent* (10th and 25th quantile for group 1) and *Trend* (90th quantile for group 2).

**Table 11:** Estimates for CO_2_ for the two groups

Variables		Quantiles					OLS
		10th	25th	50th	75th	90th	Fixed effects
GDP	G1	0.5154***	0.5427***	0.5365***	0.5181***	0.4971***	0.5236***
	G2	0.0846***	0.1014***	0.1137***	0.1301***	0.1374***	0.1153***
GDP2	G1	− 0.0066***	− 0.0069***	− 0.0064***	− 0.0061***	− 0.0056***	− 0.0063***
	G2	− 0.0007**	− 0.0008***	− 0.0008***	− 0.0010***	− 0.0010***	− 0.0008**
Shadow	G1	− 0.7230***	− 0.8097***	− 0.7804***	− 0.7311***	− 0.7108***	− 0.7473***
	G2	0.8613***	0.8711***	0.9028***	0.9019***	0.8919***	0.8874***
Manuf	G1	0.1539***	0.1533***	0.1645***	0.1736***	0.1802***	0.1724***
	G2	0.0003	0.0011	0.0003	− 0.0010	0.0041	− 0.0010
Rent	G1	0.0067	0.0128	0.0309***	0.0519***	0.0651***	0.0348***
	G2	0.0444***	0.0535***	0.0646***	0.0781***	0.0955***	0.0700***
Urban	G1	0.2040***	0.1991***	0.1943***	0.1970***	0.2006***	0.1955***
	G2	0.0241***	0.0262***	0.0270***	0.0277***	0.0297***	0.0269***
Trend	G1	− 0.0808***	− 0.0783***	− 0.0824***	− 0.0740***	− 0.0675	− 0.0751***
	G2	− 0.0081***	− 0.0097***	− 0.0104***	− 0.0114***	− 0.113***	− 0.0150***
Constant	G1	− 11.635***	− 11.214***	− 10.843***	− 10.991***	− 11.072***	− 11.135***
	G2	− 0.6538**	− 0.6570**	− 0.6407**	− 0.5908**	− 0.6244**	− 0.5516*

The Stata function *xtscc* was used for the fixed-effects estimates, and the Stata functions *xtreg* and *qreg* were used in the first and second steps of the quantile regression estimation. G1 and G2 represent group 1 and group 2, respectively

^***^, **, and * denote statistical significance at the 1%, 5%, and 10% levels, respectively

Appendix B shows the fixed effects and quantile estimates for the full sample (Table 12) and the two groups (Table 13), when we use dummy variables to control for outliers. These new estimates show slight changes relative to the previous ones, which asserts the robustness of our results.

### Panel smooth transition regression

**Fig. 2:**
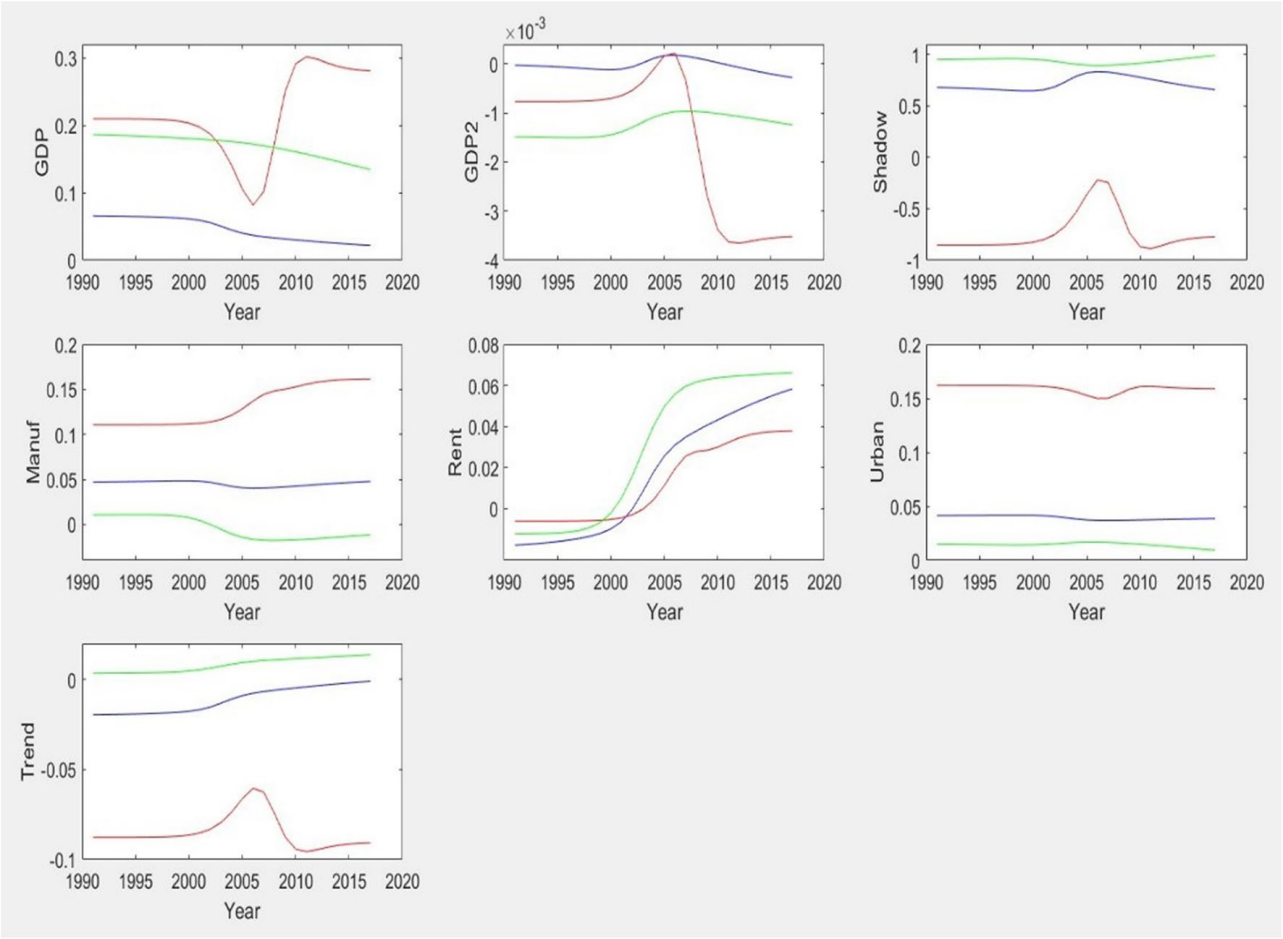
The evolution of the covariates’ slopes over time implied by the PSTR estimates. The blue line represents the full sample, the red line corresponds to the first group and the green line to the second group

Figure 2 depicts the evolution of the covariates’ slopes over time, implied by the PSTR estimates. In all cases, three regimes were chosen (*r* = 2). The linearity and no remaining heterogeneity test results and the final model estimates are available from the authors on request. For the complete sample (blue line), we observe a weakening tendency for the linkage between CO_2_ emissions and *GDP*, whereas the impact of fossil fuel rents strengthens over time, which is consistent with hypothesis 4. The other slopes remain broadly stable. For group 2 (green line), we detect the same tendencies for *GDP* and *Rent*, but the environmental degradation caused by the manufacturing share disappeared in the most recent years. Indeed, the direct mitigating effect of variable *Trend* (i.e., the effect of time not mediated by changes in other covariates' slopes), identified in Table 9, completely vanishes. The only clear patterns for group 1 (red line) are the increasing impacts of manufacturing and fossil fuel rents. Moreover, unlike group 2, the effect of time on CO_2_ emissions was more pronounced and showed a decreasing intensity in the middle of the 2000s. It does not operate through changes in the sensitivities of CO_2_ emissions to the remaining covariates.

**Fig. 3:**
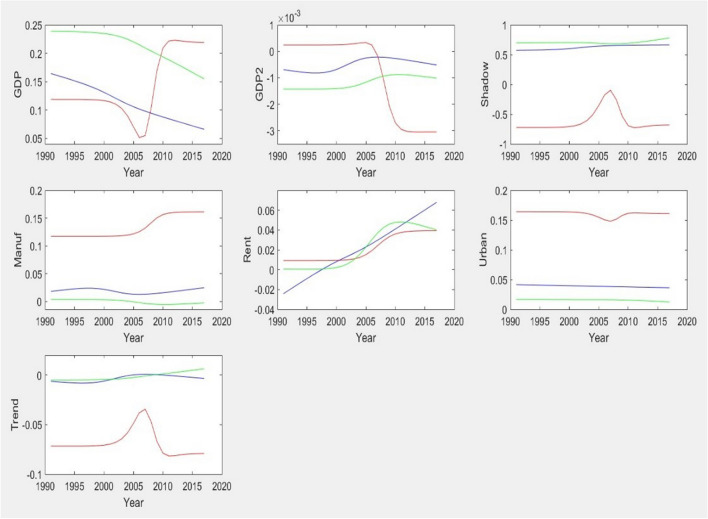
The evolution of the covariates’ slopes over time implied by the PSTR estimates with dummies. The blue line represents the full sample, the red line corresponds to the first group and the green line to the second group

Figure 3 in Appendix B shows the evolution of the estimates when we control for outliers. We only observe minor changes relative to the Fig. 2 in the main text. Thus, our conclusions remain essentially unchanged.

## Discussion

The adverse environmental impacts of the shadow economy in the second group of countries, which has lower average per capita CO_2_ emissions than group 1, are evident in all quantiles of the empirical findings, confirming our first hypothesis. Furthermore, similar results are detected by Pang et al. (2021) and Zhou (2019) in China, Baksi and Bose (2021) in developing countries, and Canh et al. (2021) but are inconsistent with Sultana et al. (2022), Chu (2022), and Sohail et al. (2021). The underlying theoretical mechanism of the noxious impacts is that the scale effect dominates the composition and technique effects. Specifically, the informal sectors mainly contain small, unlicensed agents characterized by underdeveloped or developing technologies and high CO_2_ emissions, which are hard to discover and regulate. This matter facilitates increasing underground production, especially through manufacturing activities, and hence is considered one of the main contributors to the improper environmental pressures in such economies (Elgin and Mazhar 2013). Also, the formal sector may avoid costly rules and regulations by outsourcing parts of the production process to the informal sectors. In addition, the informal transportation sector also leads to huge exhaust emissions in this group due to inefficient vehicles in clandestine transportation activities, which often hardly satisfy environmental standards (Zhou 2019).

In contrast, in the first group of countries, which includes most countries with a smaller average size of their informal economies relative to GDP, the adverse effects of the informal economy turn into favorable effects that enhance the environmental quality (Bano et al. 2018). This finding is consistent with Chu (2022). It can be justified by the fact that the composition or technique effect dominates the scale effect in the countries with less participation in the underground economy, which have higher levels of institutional quality and government effectiveness. In low environmentally degraded economies, the stringent environmental regulations and policies cause the informal sectors to protect the environment rather than pollute it. This process relates to less capital-intensive techniques, making the informal sector less motivated to participate in environmental destruction (Elgin and Oztunali 2014).

Moreover, from the left to the right tail of the emissions’ conditional distribution in each group of countries, the shadow economy’s positive or negative effects on CO_2_ emissions are relatively unchanged in intensity. Meanwhile, the full sample results indicate the overall positive response of CO_2_ emissions in reaction to the development of the shadow economy, which shows the dominant role of the countries with the greater average size of their informal economies relative to the gross domestic product in explaining CO_2_ emissions. It means that the scale effect neutralizes the appropriate impacts of the composition effect and technique effect across the full sample. The potential explanations to justify this relationship are as follows: first, technological development and decreasing CO_2_ emissions associated with underground production need considerable private and public investment. The informal sectors may not have enough financial resources for efficient technological transformation, especially through manufacturing activities requiring long-term and large-scale investment to capture the benefits (Elgin and Mazhar 2013). Second, technology transfer associated with switching from the scale effect toward the composition and technique effect requires the connectedness network to share and adopt knowledge, which entails institutional development and formal procedures (Lassa 2019). Third, the higher levels of informality, which hold for individual participants and enterprises that run outside the institutional framework, make them uninterested in engaging in this formal process to keep away from the government’s regulations. Last, human capital is a major factor affecting the adoption level of environmental innovations (De Marchi and Grandinetti 2013). However, the informal economy does not support and facilitate human capital capacity building regarding research, development, and innovative technologies.

Accordingly, the higher quality of government regulation significantly intensifies the government’s ability to establish procedures for environmental protection (Ortiz et al. 2022). Therefore, switching from underground activities to the formal economy relates to lower environmental degradation, ceteris paribus. This finding may motivate environmental policymakers to focus more on the policies related to the shadow economy.

Besides the common elements examined in the previous literature with similar viewpoints on structural, technological, and economic effects, this paper introduces the shadow economy and Trend as the significant determinants of pollution. Also, we compare how the interaction between these two factors affects *CO*_*2*_ emissions across two groups of countries with different levels of environmental regulations and technologies. To the best of our knowledge, the previous studies (Chu 2022; Adekoya et al. 2022; Alvarado et al. 2021; Deng et al. 2020; Wang et al. 2019; Canh 2019; Shahbaz et al. 2017) focused on the production process as the key factor affecting pollution and on how the structural and technological changes helped to reduce emissions. In this regard, the upward impacts of manufacturing value-added and fossil fuel rents on the emissions in group 1 are just the clear patterns in this study, corroborating our second hypothesis (Fig. 2).

Our study confirms the scale effect in group 2 (Pang et al. 2021; Zhou 2019; Baksi and Bose 2021; Canh et al. 2021; Wang and Wei 2020; Dinda 2004), while the composition and technique effects in group 1 (Chu 2022; Bano et al. 2018). As the other contribution of this paper, the suggested outcome shows that innovation and technological progress contribute to the mitigation of environmental degradation (Sultana et al. 2022; Chu (2022);; Sohail et al. 2021; Ulucak 2021; Hussain & Dogan 2021; Hussain et al. 2020), which confirms our third hypothesis. Particularly, it is driven by the adoption of the escalation of the industrialized energy system, energy efficiency, the policies attracting investment for new and renewable energy sources, and the technological and structural advancement for exhaust gas emissions reduction, which is in line with studies by Su et al. (2022), Zhou (2019), Wang et al. (2016), and Zhang and Lin (2012).

Notably, the average size of technological improvement in both sub-groups does not seem large enough to significantly support the shadow economy’s proper impact and considerably reduce environmental concerns. Therefore, weak institutional and corrupted economies should avoid deepening the shadow economy due to stricter environmental terms and regulations (Rafique et al. 2021; Chaudhuri & Mukhopadhyay 2006; Fleming et al. 2000).

Consequently, structural changes and technological improvement are suggested to dominate the improper impacts of scale effect and capture the benefits of the composition and technique effects by the economies, lowering the CO_2_ emissions. Also, research and development projects, innovation, and technological improvement can lead to environment-friendly production processes and techniques across urban areas. These proceedings can mitigate the adverse environmental effects of growing urban infrastructure and contribute to fewer CO_2_ emissions.

Moreover, explicitly and implicitly, policies to switch from a carbon-based fuel portfolio to new and renewable energy technologies will impact emissions reduction. Specifically, the explicit impact relates to the non-carbon-based fuel portfolio, while the implicit effect refers to reduced rent for fossil fuels. Besides, efficient government performance to promote environmental regulations and shrink the size of the informal economy helps the countries increase the environmental benefits achieved by the structural, technological, and resource portfolio policies, especially in developing and underdeveloped economies.

## Conclusion and policy implications

The linkage between the shadow economy and carbon dioxide (CO2) emissions was evaluated for 145 countries from 1991 to 2017. In assessing the effect of the shadow economy on CO2 emissions, the estimation methods of Canay’s (2011) panel quantile regression, panel fixed effects, and panel smooth transition regression were used. In addition, to deal with the problem of parameter heterogeneity, we used the procedure of Lin and Ng (2012) to identify groups of countries that share homogeneous parameters.

Identifying groups of countries that share homogeneity of parameters is an advised procedure when we are researching the impact of the shadow economy on the environmental degradation of the panel of countries. Indeed, this research reveals important differences in the impact of variables, otherwise masked if estimation was done for the panel of countries. For example, no environmental Kuznets curve was found for all countries. Nevertheless, we found that this environmental Kuznets curve has statistically significant parameters for the groups of countries with homogeneous parameters. This result supports different turning points for different groups of countries.

The procedure of Lin and Ng (2012) identified two groups sharing parameter homogeneity. Group 1 comprises 42 countries and includes most countries of the European Union plus the UK and the largest developed economies, such as the US and Japan. Group 2 comprises 103 countries. However, the two groups also reveal differences, as the average per capita CO2 emissions of Group 1 countries are higher than those of Group 2. Furthermore, group 1 is richer, has a higher informal economy dimension, a smaller relative average GDP, a more urban population, and a slightly higher manufacturing share of GDP. Nevertheless, the most sizable difference between the groups regards fossil fuel rents for group 1 more than triple that of group 2.

Shadow economy was revealed to contribute to reducing CO_2_ emissions in group 1 and aggravate it in group 2. Manufacturing value-added as a percentage of GDP was only statistically significant for the countries of group 1. Fossil fuel rents as a percentage of GDP aggravate the CO2 emissions, but more markedly in group 2 countries. Urban population as a percentage of the total population contributes to the aggravation of CO_2_ emissions in both groups of countries, but much more intensely for group 1. We also found evidence of a tendency to decrease CO_2_ emissions, reflecting efficiency gains over time.

The evolution over time of the covariates’ slopes implied by the PSTR estimates for the complete sample supports a weakening tendency for the linkage between CO_2_ emissions and *GDP* and an impact of fossil fuel rents strengthening over time (hypothesis 4). The same tendencies for *GDP* and fossil fuel rents were detected for the groups, but the environmental degradation caused by the manufacturing share has disappeared in recent years. The direct mitigating effect of time did not result from changes in other covariates’ slopes entirely fading away. For group 1, an increasing impact of manufacturing and fossil fuel rents on CO_2_ emissions was found. For group 2, the effect of time on CO_2_ emissions is more pronounced and shows a decrease in intensity in the middle of the 2000s.

Moreover, the multilateral impacts of the informal economy on the broad concept of environmental quality ask for interdisciplinary research. Furthermore, it is due to a wide range of hidden aspects through underground activities that can significantly intensify undesirable market effects and environmental concerns, e.g., methane emissions and marine pollution, habitat destruction, and local anomalies, which are suggested as the major limitations of this work and hence, considered to study by further research.

We can draw several conclusions from our results that may guide policymakers in their policy design. First, we have clearly shown that the shadow economy distorts the true relationship between carbon dioxide emissions and economic development. Therefore, decision-makers should strive to properly account for the informal activities in the measured gross domestic product to accurately assess their EKC stage and propose appropriate policies to reduce emissions. Second, late-developing countries may benefit from developing innovative and clean technologies. To achieve this goal, they should open their economies to foreign investment and design policies that encourage the adoption of these advanced techniques. Third, we have shown that manufacturing and urbanization contribute to environmental degradation. Countries should counteract these effects by promoting the transition to higher value-added products and services to avoid being caught in the middle-income trap and reduce emissions. They should also control and adequately plan the growth of cities to escape the environmental degradation driven by the urbanization process. Finally, countries that rely heavily on fossil fuel rents should use the resources generated by their sale to transition to a green economy. Fossil fuels are depleting resources, and these rents will not last forever. Therefore, these countries must design policies that foster sustainable development before these rents start declining.

Future research should focus on the accurate measurement of true economic activity and its relationship with environmental degradation. Countries take different approaches regarding including informal activities in the official gross domestic product reports, and the procedures adopted are often unclear, especially in less developed countries. Therefore, we think it is essential to create a comprehensive database that accurately measures the economic development of all countries to avoid distortions in assessing the EKC hypothesis.

## Data Availability

The datasets generated during and/or analyzed during the current study are available from the corresponding author upon reasonable request.

## Appendix A

### Country list

- Country list: Albania, Algeria, Angola, Argentina, Australia, Austria, Azerbaijan, Bahamas, Bahrain, Bangladesh, Belarus, Belgium, Belize, Benin, Bhutan, Bolivia, Bosnia and Herzegovina, Botswana, Brazil, Brunei Darussalam, Burkina Faso, Burundi, Cabo Verde, Cambodia, Cameroon, Canada, Chad, Chile, China, Colombia, Congo Dem. Rep., Congo Rep., Costa Rica, Cote d’Ivoire, Croatia, Cyprus, Czech Republic, Denmark, Dominican Republic, Ecuador, Egypt, El Salvador, Equatorial Guinea, Estonia, Ethiopia, Fiji, Finland, France, Gabon, Gambia, Georgia, Germany, Ghana, Greece, Guatemala, Guinea, Guinea-Bissau, Guyana, Haiti, Honduras, Hungary, Iceland, India, Indonesia, Iran, Ireland, Israel, Italy, Jamaica, Japan, Jordan, Kazakhstan, Kenya, Korea Rep., Kuwait, Kyrgyz Republic, Lao PDR, Latvia, Lebanon, Lesotho, Liberia, Lithuania, Luxembourg, Malawi, Malaysia, Maldives, Mali, Malta, Mauritania, Mauritius, Mexico, Moldova, Mongolia, Morocco, Mozambique, Myanmar, Namibia, Nepal, Netherlands, New Zealand, Nicaragua, Niger, Nigeria, Norway, Oman, Pakistan, Papua New Guinea, Paraguay, Peru, Philippines, Poland, Portugal, Qatar, Romania, Russian Federation, Rwanda, Saudi Arabia, Senegal, Sierra Leone, Singapore, Slovak Republic, Slovenia, South Africa, Spain, Sri Lanka, Suriname, Sweden, Switzerland, Tajikistan, Tanzania, Thailand, Togo, Tunisia, Turkey, Uganda, Ukraine, United Arab Emirates, UK, US, Uruguay, Venezuela R. B., Vietnam, Yemen Rep., Zambia, and Zimbabwe.
- Group 1: Angola, Azerbaijan, Belarus, Belgium, Brunei Darussalam, Chad, China, Congo Rep., Croatia, Denmark, Estonia, Fiji, Finland, France, Germany, Iceland, Iran, Ireland, Japan, Latvia, Malaysia, Maldives, Malta, Mauritania, Mongolia, Netherlands, Oman, Poland, Qatar, Romania, Russian Federation, Saudi Arabia, Singapore, Slovenia, Spain, Sweden, Ukraine, UK, US, Venezuela R. B., Vietnam, and Yemen Rep.
- Group 2: Albania, Algeria, Argentina, Australia, Austria, Bahamas, Bahrain, Bangladesh, Belize, Benin, Bhutan, Bolivia, Bosnia and Herzegovina, Botswana, Brazil, Burkina Faso, Burundi, Cabo Verde, Cambodia, Cameroon, Canada, Chile, Colombia, Congo Dem. Rep., Costa Rica, Cote d’Ivoire, Cyprus, Czech Republic, Dominican Republic, Ecuador, Egypt, El Salvador, Equatorial Guinea, Ethiopia, Gabon, Gambia, Georgia, Ghana, Greece, Guatemala, Guinea, Guinea-Bissau, Guyana, Haiti, Honduras, Hungary, India, Indonesia, Israel, Italy, Jamaica, Jordan, Kazakhstan, Kenya, Korea Rep., Kuwait, Kyrgyz Republic, Lao PDR, Lebanon, Lesotho, Liberia, Lithuania, Luxembourg, Malawi, Mali, Mauritius, Mexico, Moldova, Morocco, Mozambique, Myanmar, Namibia, Nepal, New Zealand, Nicaragua, Niger, Nigeria, Norway, Pakistan, Papua New Guinea, Paraguay, Peru, Philippines, Portugal, Rwanda, Senegal, Sierra Leone, Slovak Republic, South Africa, Sri Lanka, Suriname, Switzerland, Tajikistan, Tanzania, Thailand, Togo, Tunisia, Turkey, Uganda, United Arab Emirates, Uruguay, Zambia, and Zimbabwe.

## Appendix B

### Estimates with dummy variables

**Table 12:** Estimates for CO_2_ with dummies

Variables	Quantiles					OLS
	10th	25th	50th	75th	90th	Fixed effects
GDP	− 0.0084	− 0.0282***	− 0.0033	0.0310***	0.0386***	− 0.0155
GDP2	− 0.0007***	− 0.0004***	− 0.0005***	− 0.0007***	− 0.0007***	− 0.0004
Shadow	0.8402***	0.9384***	0.9102***	0.8909***	0.8159***	0.9330***
Manuf	0.0224***	0.0227***	0.0182***	0.0215***	0.0399***	0.0272***
Rent	− 0.0063	0.0158***	0.0207***	0.0371***	0.0623***	0.0249***
Urban	0.0545***	0.0577***	0.0580***	0.0586***	0.0619***	0.0577***
Trend	− 0.0161***	− 0.0173***	− 0.0190***	− 0.0192***	− 0.0167***	− 0.0204***
Constant	− 0.9229***	− 0.9451***	− 0.7526***	− 0.7800***	− 0.9132***	− 0.7909**

The Stata function *xtscc* was used for the fixed-effects estimates, and the Stata functions *xtreg* and *qreg* were used in the first and second steps of the quantile regression estimation

^***^, and ** denote statistical significance at the 1%, and 5% levels, respectively

**Table 13:** Estimates for CO_2_ for the two groups with dummies

Variables		Quantiles					OLS
		10th	25th	50th	75th	90th	Fixed effects
GDP	G1	0.4845***	0.5056***	0.5158***	0.5217***	0.4956***	0.5131***
	G2	0.0388***	0.0642***	0.0947***	0.1091***	0.1144***	0.0911**
GDP2	G1	− 0.0062***	− 0.0063***	− 0.0062***	− 0.0060***	− 0.0056***	− 0.0062***
	G2	− 0.0004***	− 0.0006***	− 0.0007***	− 0.0008***	− 0.0008***	− 0.0007**
Shadow	G1	− 0.7615***	− 0.7932***	− 0.7234***	− 0.7988***	− 0.8456***	− 0.7440***
	G2	0.8912***	0.9349***	0.9177***	0.9372***	0.9400***	0.9071***
Manuf	G1	0.1254***	0.1202***	0.1555***	0.1498***	0.1412***	0.1653***
	G2	0.0003	0.0028***	0.0051***	0.0030***	0.0041*	0.0037
Rent	G1	0.0021	0.095**	0.0262***	0.0437***	0.0472***	0.0287***
	G2	0.0271***	0.0360***	0.0337***	0.0503***	0.0752***	0.0429***
Urban	G1	0.2020***	0.1972***	0.1923***	0.1984***	0.1975***	0.1941***
	G2	0.0232***	0.0261***	0.0264***	0.0275***	0.0317***	0.0266***
Trend	G1	− 0.0806***	− 0.0840***	− 0.0829***	− 0.0824***	− 0.0680	− 0.0786***
	G2	− 0.0055***	− 0.0086***	− 0.0090***	− 0.0100***	− 0.0110***	− 0.0133***
Constant	G1	− 10.740***	− 10.160***	− 10.493***	− 10.464***	− 9.667***	− 10.777***
	G2	− 0.2867***	− 0.4654***	− 0.5061***	− 0.5056***	− 0.6125***	− 0.4109*

The Stata function *xtscc* was used for the fixed-effects estimates, and the Stata functions *xtreg* and *qreg* were used in the first and second steps of the quantile regression estimation. G1 and G2 represent group 1 and group 2, respectively

^***^, **, and * denote statistical significance at the 1%, 5%, and 10% levels, respectively

## Footnotes

1 International Resource Panel (2019) reports that the extraction and processing of natural resources account for approximately 50% of the world’s greenhouse gas emissions. Furthermore, the report notes that the resource-associated effects on water stress as well as biodiversity loss because of land use are considerable (Baloch et al. 2019).

2 Among the developed OECD countries, New Zealand and Japan do not include estimates of the non-observed economy in their national accounts (United Nations Economic Commission for Europe 2008).

3 Eurostat breaks down the informality type into seven categories: N1—underground producer; N2—illegal producer, not registered; N3—producer is not obliged to register; N4—registered legal person not included in statistics; N5—registered entrepreneur not registered in statistics; N6—misreporting by the producer; N7—statistical deficiencies in the data (Gyomay, 2014).

4 We only present the results of the preliminary tests for the complete sample. Separate tests for the two groups are available from the author on request.
